# Tropomyosin 3 (TPM3) function in skeletal muscle and in myopathy

**DOI:** 10.1186/s13395-023-00327-x

**Published:** 2023-11-07

**Authors:** Matthias R. Lambert, Emanuela Gussoni

**Affiliations:** 1 Division of Genetics and Genomics, Boston Children’s Hospital, 300 Longwood Ave., Boston, MA 02115 USA; 2 Department of Pediatrics, Harvard Medical School, Boston, MA 02115 USA; 3 The Stem Cell Program, Boston Children’s Hospital, Boston, MA 02115 USA

**Keywords:** Skeletal muscle, Tropomyosin, TPM3, Thin filaments, Congenital myopathy, Rare diseases

## Abstract

The tropomyosin genes (*TPM1-4*) contribute to the functional diversity of skeletal muscle fibers. Since its discovery in 1988, the *TPM3* gene has been recognized as an indispensable regulator of muscle contraction in slow muscle fibers. Recent advances suggest that TPM3 isoforms hold more extensive functions during skeletal muscle development and in postnatal muscle. Additionally, mutations in the *TPM3* gene have been associated with the features of congenital myopathies. The use of different in vitro and in vivo model systems has leveraged the discovery of several disease mechanisms associated with TPM3-related myopathy. Yet, the precise mechanisms by which TPM3 mutations lead to muscle dysfunction remain unclear. This review consolidates over three decades of research about the role of TPM3 in skeletal muscle. Overall, the progress made has led to a better understanding of the phenotypic spectrum in patients affected by mutations in this gene. The comprehensive body of work generated over these decades has also laid robust groundwork for capturing the multiple functions this protein plays in muscle fibers.

## Background

**Fig. 1:**
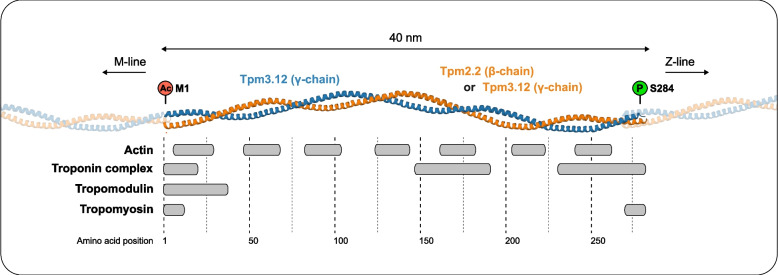
Structure of striated tropomyosin and interactions. The tropomyosin molecule (284–285 amino acids) is an α-helical chain that self-assembles into parallel coiled-coil homo or heterodimers. This structure polymerizes head-to-tail to form an uninterrupted coiled-coil along the entire length of actin filaments in both muscle and non-muscle cells. In skeletal muscle, striated isoforms preferentially form heterodimers: Tpm1.1/Tpm2.2 (i.e., αβ dimers) in fast myofibers and Tpm2.2/Tpm3.12 (i.e., γβ dimers) in slow myofibers. The structural organization of tropomyosin is essential for its association with actin filaments, made of weak yet specific electrostatic interactions which enable tropomyosin to shift between regulatory states with a low energy cost. Each striated tropomyosin molecule spans the length of seven consecutive actin monomers. Putative binding regions for actin [14], subunits of the troponin complex [15–17], tropomodulin (Tmod) [18], tropomyosin [19], as well as myosin (not shown in the figure) [20], leiomodin (Lmod) (not shown in the figure) [21], and nebulin (not shown in the figure) [22], have been proposed on tropomyosin. N-terminal acetylation (Methionine 1) and C-terminal phosphorylation (serine 284) enhance head-to-tail interaction between neighboring tropomyosin and increase affinity to actin. As we write this review, there exists no atomic resolution of full-length tropomyosin structure. Most studies have used α-chain fragments encoded by the *TPM1* gene to decipher the design of the tropomyosin molecule. Furthermore, tropomyosin can adopt different conformations implying binding changes, which have not yet been fully resolved either by crystallography or molecular dynamics (MD) simulation. Despite the high similarity among tropomyosin isoforms, the nature and precise location of putative binding sites on TPM3 isoforms remain in debate

Tropomyosin consists of a two-chained, α-helical coiled-coil protein, which polymerizes head-to-tail to form a continuous strand along the entire length of actin filaments (for review about tropomyosin structure, see [1]) (Fig. 1). This structural organization serves as a gatekeeper, controlling the recruitment and activity of several actin-binding partners (e.g., myosin, non-muscle myosin, troponin, tropomodulin, Arp2/3, ADF/cofilin, gelsolin), and ultimately regulates the function and stability of actin filaments [1, 2]. The tropomyosin genes (*TPM1*, *TPM2*, *TPM3*, and *TPM4*) encode several isoforms classified into two categories: low molecular weight isoforms (LMW, cytoskeletal isoforms) and high molecular weight isoforms (HMW, striated isoforms) (for systematic nomenclature, see [3]). Most isoforms exhibit distinct temporal and tissue-specific expression, non-redundant function and non-overlapping localization that contribute to the functional diversity of actin filaments [2, 4, 5]. Initially discovered as a component of myofibrils [6, 7], striated tropomyosin isoforms incorporate into actin thin filaments in skeletal muscle and cardiac tissues [8, 9], playing an essential role in the regulation of muscle contraction [10, 11]. In skeletal muscle, striated tropomyosin isoforms also contribute to the functional diversity of muscle fibers. For instance, the α-tropomyosin isoform (Tpm1.1) encoded by the *TPM1* gene is exclusively expressed in fast muscle fibers (type 2), while the striated β-tropomyosin isoform (Tpm2.2) encoded by the *TPM2* gene is expressed in both slow (type 1) and fast muscle fibers [12]. The striated γ-tropomyosin isoform (Tpm3.12) encoded by the *TPM3* gene is exclusively expressed in slow muscle fibers [3, 13]. In contrast, cytoskeletal tropomyosin isoforms are ubiquitously expressed in mammalian tissues and regulate the function of a vast array of actin-based structures including skeletal muscle triad, endoplasmic and sarcoplasmic reticulum, stress fibers, endosomes, cell cortex, and Golgi-associated short filaments (for recent review about cytoskeletal isoforms, see [2]).

In humans, mutations in the *TPM2* and *TPM3* genes have been associated with congenital myopathies [23]. The term congenital myopathy refers to a genetically heterogeneous group of early-onset skeletal muscle disorders characterized by variable degrees of muscle weakness and defined by distinct structural abnormalities observed in muscle biopsies [24]. The estimated prevalence is 1 in 36,000 children worldwide [25]. Unlike muscular dystrophies, congenital myopathies have a static or slowly progressing clinical course accompanied by normal serum creatine kinase levels, suggesting the preservation of sarcolemmal integrity in muscle fibers. Additionally, there exists a large spectrum of clinical manifestations ranging from mild to severe and sometimes fatal cases attributable to respiratory and cardiac insufficiency. Significant advances in understanding the genetic basis of congenital myopathies have resulted in the discovery of mutations in more than 20 genes [26]. Most of these genes have been shown to encode proteins involved in calcium homeostasis, excitation–contraction coupling, filament assembly, protein turnover, as well as membrane trafficking and remodeling [27]. Several promising therapeutic strategies are currently under development for some of these conditions [27].

TPM3-related myopathy was first described in a large Australian family in 1992 [28, 29]. Since then, several case reports have been published, yet the molecular mechanisms responsible for TPM3-related myopathy remain poorly understood. For years, the study of TPM3-related myopathy received little attention. So far, there exists no cure or ongoing clinical trial for this condition. There exists a pressing need to improve the diagnosis of TPM3-related myopathy, understand the molecular causes of the disease, and develop adequate treatments for the patients. In this review, we will provide an update on TPM3 function in skeletal muscle and the current state of TPM3-related myopathy.

## TPM3 function in skeletal muscle

### The *TPM3* gene

**Fig. 2:**
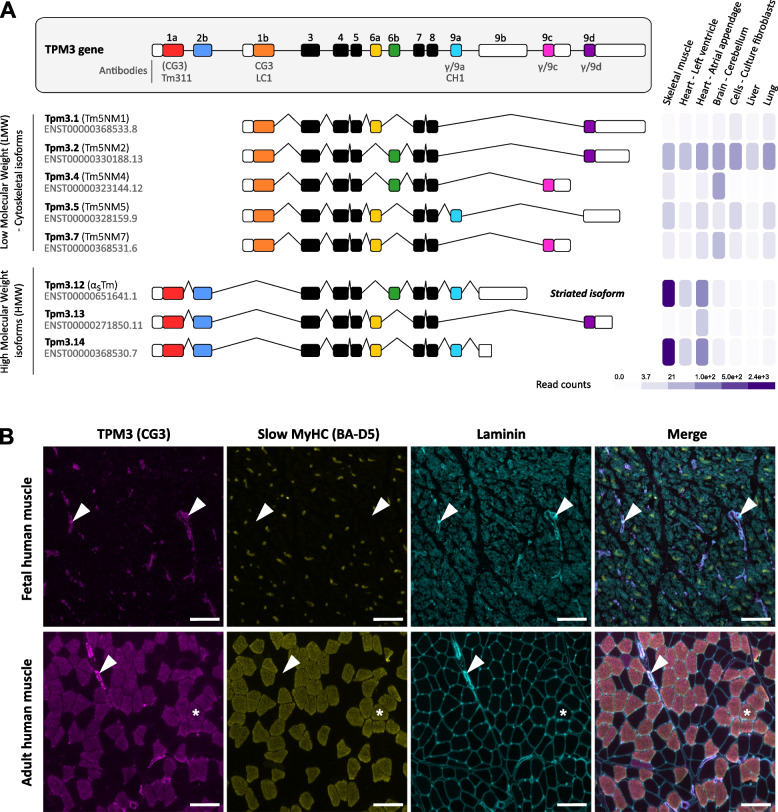
Structure of the human *TPM3* gene and tissue expression. **A** Schematic representation of the human *TPM3* gene and isoforms. Colored boxes represent the coding exons from 1 to 9, white boxes represent untranslated 5’ and 3’ UTR sequences, and lines represent the introns. Isoforms generated by the *TPM3* gene can be classified into two categories: Low Molecular Weight isoforms (LMW, cytoskeletal isoforms) starting from exon 1b and High Molecular Weight isoforms (HMW) starting from exon 1a. The nomenclature (Tpm3.X) follows the recommendation provided by Geeves et al. 2015, *J Muscle Res Cell Motil* [3]. The previous nomenclature is also indicated in brackets. Exon-specific antibodies used to detect the TPM3 isoforms are indicated underneath the respective exons (for detailed information, see Schevzov et al. 2011, *BioArchitecture* [36]). The CG3 antibody specifically detects all cytoskeletal isoforms containing exon 1b, as well as high molecular weight isoforms via cross-reaction with exon 1a or 2b (indicated in brackets). On the right side, tissue-specific expression (read counts) in adult human skeletal muscle, heart (left ventricle and atrial appendage), brain (cerebellum), culture fibroblasts (cells), liver and lung is provided for each isoform (data available via gtexportal.org). **B** Immunofluorescence using CG3 antibody indicates that TPM3 protein expression can be detected in different cell types in human fetal and adult skeletal muscle. In fetal muscle, the CG3 signal co-localizes with blood vessels (arrows). In adult muscle, the CG3 co-localizes with both blood vessels (arrows) and type 1, slow muscle fibers (asterisks). The scale bar indicates 100 µm

The human *TPM3* gene (also known as the *γ-TM*, *NEM1*, *TM5,* or *TM*_*nm*_ gene) is located in the 1q21.3 region of chromosome 1 and consists of 14 exons spanning approximately 39 kb of genomic DNA [30]. Transcript analyses have identified a wide diversity of isoforms that derive from alternative promoter selection and alternative exon splicing operating on a developmental and cell-specific basis [31–34]. As of today, the human *TPM3* gene can produce up to twenty-seven transcripts [35]. The main isoforms and postnatal tissue-specific expressions are presented in Fig. 2A. The Low Molecular Weight isoforms (LWM), also commonly named non-muscle isoforms or cytoskeletal isoforms, are composed of 247–248 amino acids (28–30 kDa) starting from exon 1b. These include Tpm3.1, Tpm3.2, Tpm3.4, Tpm3.5, and Tpm3.7 isoforms that differ from the alternative splicing of exons 6a/6b and C-terminal exons 9a/9c/9d. The Tpm3.2 and Tpm3.5 isoforms are ubiquitously expressed across human postnatal tissues while Tpm3.4 and Tpm3.7 isoforms (both containing exon 9c) show tissue-specific expression in brain. A second group consists of High Molecular Weight isoforms (HMW) composed of 285 amino acids (34 kDa) starting from exon 1a. These include Tpm3.12, Tpm3.13, and Tpm3.14 isoforms. The Tpm3.12 isoform (also known as γ-TPM, αTPM_slow_, α_S_TPM, or striated isoform) is highly expressed in postnatal skeletal muscle tissues. However, this broad diversity of tropomyosin isoforms and the high degree of similarity between the four tropomyosin genes make the detection of individual isoform expression at the protein level very challenging. In this context, exon-specific antibodies have constituted a valuable resource to demonstrate tissue- and cell-specific expression of a subset of tropomyosin isoforms in multiple model systems (for review, see [36]). Antibodies that react with the TPM3 isoforms are also listed in Fig. 2A. The CG3 antibody is known to detect all the products generated by the *TPM3* gene in mouse and human tissues [36, 37]. In human fetal skeletal muscle, the CG3 antibody predominantly stains the blood vessels, while in adult human skeletal muscle, the CG3 antibody stains both blood vessels and myofibers positive to slow myosin heavy chain (MyHC) antibody (Fig. 2B). Despite the development of exon-specific antibodies, our knowledge about the developmental as well as the tissue- and cell-specific expression of TPM3 protein isoforms is still very limited. The combination of exon-specific antibodies with other techniques such as in situ hybridization, RNA sequencing, and top-down proteomics have nonetheless been instrumental to, at least partially, address TPM3 isoform-specific expression as detailed below.

### Isoform-specific expression during skeletal muscle development and in postnatal muscles

**Fig. 3:**
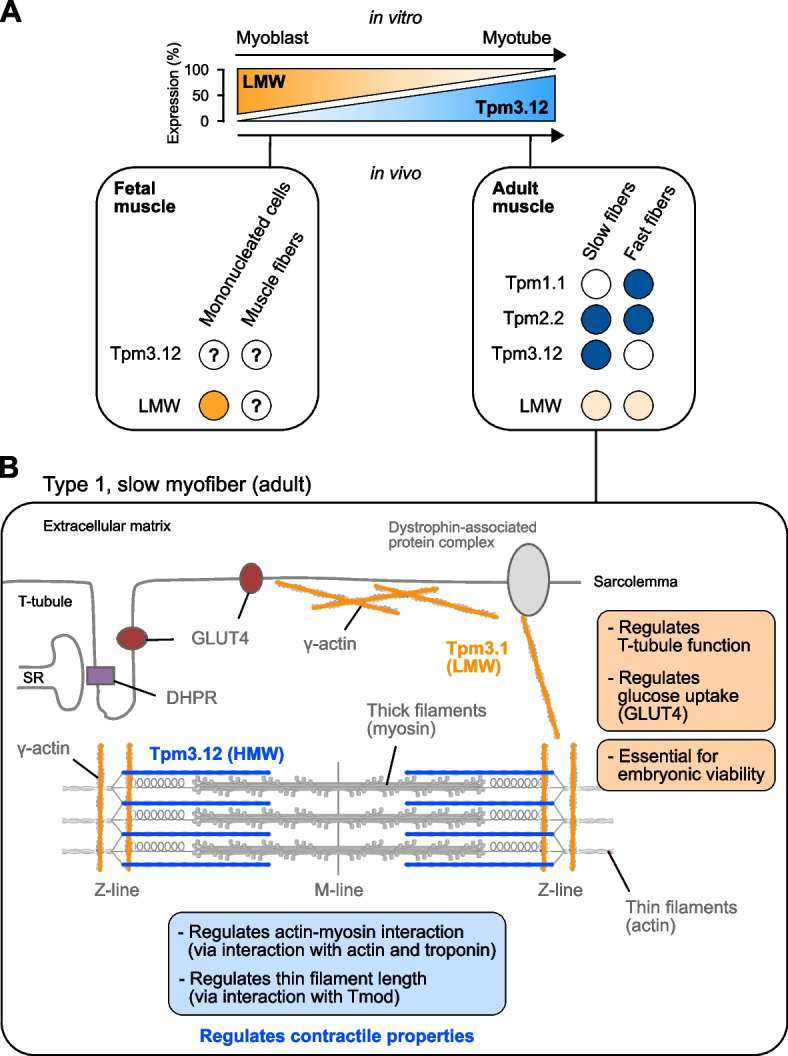
Temporal and spatial expression of TPM3 isoforms in skeletal muscle. **A** Cytoskeletal (i.e., low molecular weight isoforms, LMW) and striated isoforms (i.e., Tpm3.12) show opposite expression patterns during myogenic differentiation (in vitro) and skeletal muscle development (in vivo). Additionally, striated tropomyosin isoforms show fiber-type specific expression in postnatal muscles. Striated Tpm3.12 isoform is exclusively expressed in postnatal slow muscle fibers. In contrast, striated Tpm1.1 isoform (encoded by the *TPM1* gene is exclusively expressed in fast muscle fibers. The striated Tpm2.2 isoform (encoded by the *TPM2* gene) is expressed in both slow and fast muscle fibers. The low molecular weight isoforms (LMW) are expressed at a low level in both slow and fast muscle fibers. **B** Cytoskeletal and striated isoforms also show different sub-localizations in muscle fibers. The striated Tpm3.12 isoform incorporates into actin thin filaments at the sarcomere in slow muscle fibers. In contrast, the cytoskeletal Tpm3.1 isoform incorporates into γ-actin in both subsarcolemmal area and region adjacent to the Z-line, and co-localized with glucose transporter GLUT4 and T-Tubule constituent DHPR. Abbreviations: SR: sarcoplasmic reticulum

During skeletal muscle development, several studies have pointed out that cytoskeletal and striated isoforms have opposite expression patterns (Fig. 3A). Cytoskeletal TPM3 isoforms are highly expressed very early on during embryonic development and are essential for embryonic viability in mice and survival of embryonic stem cells [38, 39]. Interestingly, expression of cytoskeletal isoforms including the Tpm3.1 isoform tends to progressively decrease as muscle development progresses (Fig. 3A) [40–42]. Recent single-cell transcriptomics have shown that *TPM3* gene expression can be detected in different cell populations during the development of human hindlimb muscles and myogenic differentiation in vitro including some hematopoietic lineages (SRGN^+^), endothelial cells (ESAM^+^), tenogenic cells (TNMD^+^), mesenchymal population (PDGFRA^+^, THY1^+^), chondrogenic cells (SOX9^+^, COL2A1^+^), Schwann cells (CDH19^+^), neurons (DCX^+^), smooth muscle cells (MYLK^+^), myocytes (MYOG^+^, MYH3^+^), and to a lesser extent in myogenic progenitor cells (PAX3^+^, PAX7^+^) [41]. However, the differential expression of TPM3 isoforms between cell populations remains largely unknown. In contrast, the striated Tpm3.12 isoform shows very low expression at both transcript and protein levels and represents a relatively small amount (around 3%) of total striated tropomyosin (i.e., Tpm1.1, Tpm2.2, Tpm3.12) during fetal development of mouse and human skeletal muscle [13, 40, 43]. This inverse relationship between cytoskeletal Tpm3.1 and striated Tpm3.12 isoform expression was also observed in human primary and C2C12 proliferating myoblasts in culture (Fig. 3A) [40].

Unlike cytoskeletal isoforms, expression of striated Tpm3.12 isoform significantly increases by at least 5-fold during the perinatal period and can account for one-third of total striated tropomyosin [13, 40, 43, 44]. This abrupt increase occurs around week 36 of gestation in humans [43], or during the first weeks after birth in mice [13]. Studies performed in humans, mice, bovines, and zebrafish show that Tpm3.12 then correlates with slow myosin heavy chain (sMyHC) expression in skeletal muscle [13, 37, 45–47]. Mass-spectrometry revealed that Tpm3.12 is specifically expressed in slow-MyH7^+^ myofibers [45]. In contrast, the striated isoform generated by the *TPM1* gene (i.e., Tpm1.1) is restricted to myofibers expressing fast-MyHC, while the striated isoform generated by the *TPM2* gene (i.e., Tpm2.2) is present in equal amount in both slow and fast myofibers (Fig. 3A) [45]. Conversely, the presence of Tpm3.12 isoform co-expressed with slow/cardiac-MyHC at the protein level in cardiac tissue remains unclear [34, 48, 49]. If present, Tpm3.12 would account for less than 10% of total striated tropomyosin since the TPM1 striated isoform represents the predominant tropomyosin expressed in the heart.

Several studies have shown that the regulation of tropomyosin expression, specific to muscle fiber types, occurs at various levels of gene expression. For instance, the level of CpG methylation aligns with the mutually exclusive expression of *TPM1* and *TPM3* genes in mature muscle fibers indicating the involvement of epigenetic regulation [50, 51]. The simultaneous expression of striated Tpm3.12 isoform and slow-MyHC in adult myofibers can also be explained by the intervention of transcription factors that either activate (i.e., NFAT, MEF2, PGC-1α) or repress (i.e., Sox6) the expression of genes related to slow muscle fibers [52–54]. Both regulation at epigenetic and transcriptional levels are supported by recent single-nucleus transcriptomics performed in mouse skeletal muscle tissues showing that *TPM3* pre-mRNAs are predominantly expressed in slow Myh7^+^ myonuclei as opposed to *TPM1* pre-mRNAs expressed in fast Myh4^+^ myonuclei [55, 56]. Work in zebrafish has also proposed another mechanism of regulation at the post-transcriptional level in which direct interaction of Quaking (Qki) RNA-binding proteins with 3’ UTR region of Tpm3.12 transcript is required for Tpm3.12 accumulation in slow muscle fibers [47]. Quaking (Qki) proteins have been identified as major splicing regulators during muscle development (for review, see [57]), suggesting that Qki proteins might be involved in post-transcriptional splicing of nascent TPM3 RNAs, potentially regulating the inclusion of exon 9 or exon 6b and regulating Tpm3.12 transcript abundance in slow muscle fibers. Yet, striated tropomyosin transcript level does not always correlate with protein expression level. For instance, transgenic overexpression of Tpm3.12 induces compensatory reduction of endogenous striated tropomyosin isoforms (Tpm1.1, Tpm2.2, Tpm3.12) in skeletal muscle [58, 59]. Striated tropomyosin isoforms are located at the sarcomere along the entire length of thin actin filaments (Fig. 3B) [8, 9], and it is hypothesized that translational compensation ensures that the expression level of striated tropomyosin pool remains unaltered, maintaining the strict protein stoichiometry at the sarcomere [60]. In contrast, ablation of Tpm3.1 isoform does not lead to compensatory expression of other cytoskeletal isoforms in skeletal muscle [61]. In addition to the postnatal specialization of muscle fibers, this sophisticated regulation taking place at the epigenetic, transcriptional, post-transcriptional, and post-translational levels would allow the dynamic expression of striated isoforms during muscle fiber type transitions induced by various factors such as injury, denervation, immobilization, exercise, or aging [62–64]. This would ensure the proper functioning of muscle fibers throughout these adaptative processes.

### The striated Tpm3.12 isoform specifically tunes contractile function in slow muscle fibers

The high degree of similarity among striated isoforms (86–91%) goes beyond evolutionary redundancy and holds functional significance. For instance, the substitution of Tpm1.1 (α-chain) with Tpm3.12 isoform (γ-chain) in mouse cardiac tissue leads to alterations in diastolic and systolic function, along with a reduction in myofilament Ca^2+^ sensitivity [65]. The association of tropomyosin with actin filaments is made of weak yet specific electrostatic interactions which enable tropomyosin to shift between regulatory states with a low energy cost [14]. In this context, biophysical studies reveal that striated isoforms exhibit differences in their preferred position on actin [66]. Additionally, sequence alignments reveal notable variations in both troponin-binding and tropomodulin-binding regions of tropomyosin which can have specific impacts on contractile properties and thin filament assembly (for review, see [16, 67]) (Fig. 3B). A three-state model in which tropomyosin adopts three positions on actin-based thin filaments is proposed as the basis for the steric regulation of muscle contraction (for review, see [10, 11]). In brief, calcium released from the sarcoplasmic reticulum (as a consequence of action potential and membrane depolarization) binds to troponin C. In this context, Ca^2+^-binding releases the inhibitory action of troponin I on tropomyosin location, allowing a shift of tropomyosin to partially expose myosin binding sites, and promote weak binding of myosin to actin. Myosin head interaction then moves tropomyosin 10° further on actin to promote strong myosin binding and initiate power stroke. The consequences of TPM3 mutations on tropomyosin position and muscle contraction are detailed later in this review (section “Impaired association between tropomyosin, actin and troponin”). Furthermore, correct thin filament length is required to achieve proper muscle function depending on physiological requirements. In skeletal muscle, the thin filament length is precisely specified; slow muscle fibers have longer thin filaments, while fast muscle fibers have shorter thin filaments [68]. Downregulation of Tpm3.12 isoform results in shorter actin filaments in zebrafish [47]. In contrast, the presence of Tpm3.12 results in longer actin filaments in vitro [69], suggesting a role of Tpm3.12 in the regulation of thin filament length. The binding of tropomodulin (Tmod) and leiomodin (Lmod) to tropomyosin enhances both Tmod’s pointed-end capping activity and Lmod’s nucleation activity, respectively [70, 71]. In contrast, disruption of tropomyosin-binding regions on Tmod impaired thin filament assembly [72, 73]. In this context, Tmod1 has a higher binding affinity to Tpm3.12 over Tpm1.1 and Tpm2.2 [43]. Additionally, Tpm3.12 isoform binds the actin filament with higher affinity than Tpm1.1, which may influence the maintenance of thin filaments length and integrity through cofilin 2 [69, 74, 75]. In skeletal muscle, striated isoforms have the ability to either form homo- or heterodimers with αβ dimers (in fast myofibers) and γβ dimers (in slow myofibers) being predominantly found. However, the composition of dimer populations can significantly vary across different muscle groups and play a crucial role in determining contractile properties in fiber types (for review, see [76, 77]). Dimer populations substantially differ in thermostability and affinity to actin, and differentially affect the velocity of actin filaments using in vitro motility assay [78, 79]. Collectively, these findings support the conclusion that there are functional differences among the three striated isoforms and that Tpm3.12 specifically tunes the contractile function of slow muscle fibers through specific interaction with actin, troponin, and tropomodulin.

### Function of cytoskeletal isoforms in skeletal muscle

Cytoskeletal isoforms of TPM3 control the access of several actin-binding proteins including non-muscle myosin isoforms, actin depolymerizing factor (ADF)/cofilin, gelsolin, Arp2/3 complex and tropomodulin in a vast array of actin-based structures such as stress fibers, endosomes, cell cortex, post-synaptic density of dendritic spines, skeletal muscle triad (for review, see [2]). It has been shown that cytoskeletal isoforms bind actin filaments with different dynamics which correlate with distinct effects on actin-binding proteins and competitive binding [80]. Therefore, given the wide diversity of cytoskeletal isoforms, tropomyosin orchestrates the stability of distinct actin filament populations and the recruitment and activation of different myosin isoforms, and ultimately regulates a multitude of intracellular mechanisms in an isoform-dependent manner [2, 5]. For instance, Tpm3.1 isoform inhibits the Arp2/3 complex-mediated actin assembly and can recruit tropomodulin at the pointed end [81, 82]. It has also been shown that Tpm3.1 and cofilin compete for binding on actin filaments, which can influence actin disassembly [80, 83–85]. Furthermore, Tpm3.1 promotes the preferential recruitment and activation of non-muscle myosin IIA (Myo2a) to actin filaments [80, 83]. However, most of these mechanisms have been studied in vitro and in non-muscle cells, and therefore little is known about the role of cytoskeletal isoforms in skeletal muscle. In C2C12 myoblasts, the Tpm3.1 isoform is predominantly found at the cell periphery [86]. In contrast, the Tpm3.1 isoform colocalizes with γ-actin in both the subsarcolemmal area and the region adjacent to the Z-line as well as with the glucose transporter GLUT4 and the T-Tubule constituent DHPR in all muscle fibers [37, 61, 87] (Fig. 3B). In this context, ablation of the Tpm3.1 isoform has been shown to impair T-tubule morphology and function associated with altered muscle contractile properties in knockout mice [87]. Ablation and overexpression of the Tpm3.1 isoform also profoundly modify glucose uptake in skeletal muscle (as well as in white adipose tissue and heart) through the regulation of GLUT4 trafficking and fusion to the membrane [61] (Fig. 3B). In this context, Tpm3.1 promotes the recruitment of Myo2a and restricts the binding of Myo1c to actin filaments, limiting the amount of contractile force necessary for the transport and fusion of GLUT4 vesicles to the membrane [61]. Collectively, these studies indicate that the cytoskeletal isoforms of TPM3 can play important roles in skeletal muscle function, but these roles remain largely unknown.

## TPM3-related myopathy

### The clinical spectrum of TPM3-related myopathy

**Fig. 4:**
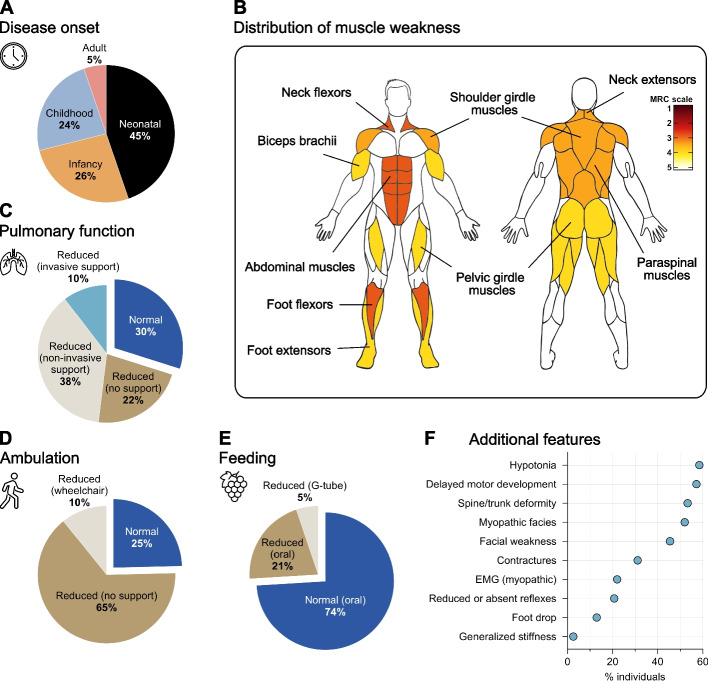
The clinical spectrum of TPM3-related myopathy. **A** Disease onset (%, *n* = 77 patients). **B** Distribution of muscle weakness based on the MRC scale (*n* = 10 patients). **C** Status of pulmonary function (%, *n* = 77 patients). **D** Status of ambulation (%, *n* = 65 patients). **E** Status of feeding function (%, *n* = 77 patients). **F** Additional features commonly observed in patients across the literature (%, *n* = 77 patients). Aggregated information from the literature has been used to generate this figure (references [23, 28, 29, 43, 46, 88–126] as of August 2023)

TPM3-related myopathy (OMIM #191030) is a subtype of congenital myopathy caused by mutations in the *TPM3* gene. Aggregated information from the literature regarding the clinical presentation of TPM3-related myopathy is presented in Fig. 4. The clinical spectrum of TPM3-related myopathy is broad, ranging from mild to severe presentations. The first onset of symptoms is typically observed at birth or during infancy (70% of cases), characterized by hypotonia and delayed motor milestones along with normal cognitive development (Fig. 4A). Individuals eventually show functional improvement until reaching mild-to-late adolescence, at which point muscle function stabilizes or slowly declines [88]. In rare cases, failure to achieve motor milestones is observed, and premature deaths are documented in three families [89–95]. Conversely, childhood-onset, and to a lesser extent adult onset, are observed in 30% of cases (Fig. 4A). In TPM3-related myopathy, patients commonly develop static to slowly progressive muscle weakness in one or several muscle groups (Fig. 4B). Muscle strength testing using the MRC scale shows prominent muscle weakness in the neck flexors, abdominal muscles, and ankle dorsiflexors. Additionally, reduction in muscle strength is often observed in the neck extensors, shoulder girdle muscles, pelvic girdle muscles, and paraspinal muscles (Fig. 4B). Patients also display distinct patterns of mild muscle fatty infiltration, which could potentially serve as valuable diagnostic indicators [96–103] (for a recent review, see [104]). Despite these typical observations, there can be significant variations in the distribution of muscle weakness and the degree of inter-myofiber fat infiltration among patients. While some may exhibit generalized muscle weakness, others might display more localized weakness in axial, proximal, or distal muscles, which could partially contribute to the broad spectrum of clinical manifestations observed in TPM3-related myopathy.

Reduction in pulmonary function (e.g., reduced force vital capacity) is a major feature observed in patients suggesting weakness of respiratory muscles including the diaphragm (Fig. 4C) [127]. This often requires nocturnal ventilation with bilevel positive airway pressure (BiPAP), even for patients that remain fully ambulant (48% with ventilatory support vs. 10% with wheelchair) (Fig. 4C, D). In severe cases, invasive ventilation usually starts within the first decade of life, while in typical cases, non-invasive ventilation begins in the second or third decade. Childhood onset and adult-onset cases, on the other hand, typically begin ventilation during the third decade of life or even later. In a number of cases, untreated respiratory muscle insufficiency was life-threatening and led to respiratory distress and/or heart failure regardless of disease severity and disease onset [23, 88, 89, 93, 95, 96, 100, 105, 106].

Although most patients maintain their ability to walk, approximately 70% of them experience impaired ambulation (e.g., cannot run, cannot jump, slow runner, short distance walk, stairs difficult) (Fig. 4D). Furthermore, about 25% of patients have feeding difficulties (e.g., poor suck during infancy, dysphagia) and 5% require G-tube placement (Fig. 4E). Additional features such as spine and chest deformities (e.g., scoliosis, lumbar lordosis), myopathic facies (e.g., high-arched palate, long narrow face), facial weakness (e.g., ptosis), foot drop, reduced reflexes, poor muscle bulk and normal serum CK level are commonly observed in patients (Fig. 4F). Mild contractures, and joint hypermobility are also documented in some patients, while generalized muscle stiffness associated with severe reduction in joint mobility was observed in two families [92]. Conversely, extraocular muscles and cardiac function are frequently spared in TPM3 patients.

### The spectrum of histological findings in muscle biopsies

**Fig. 5:**
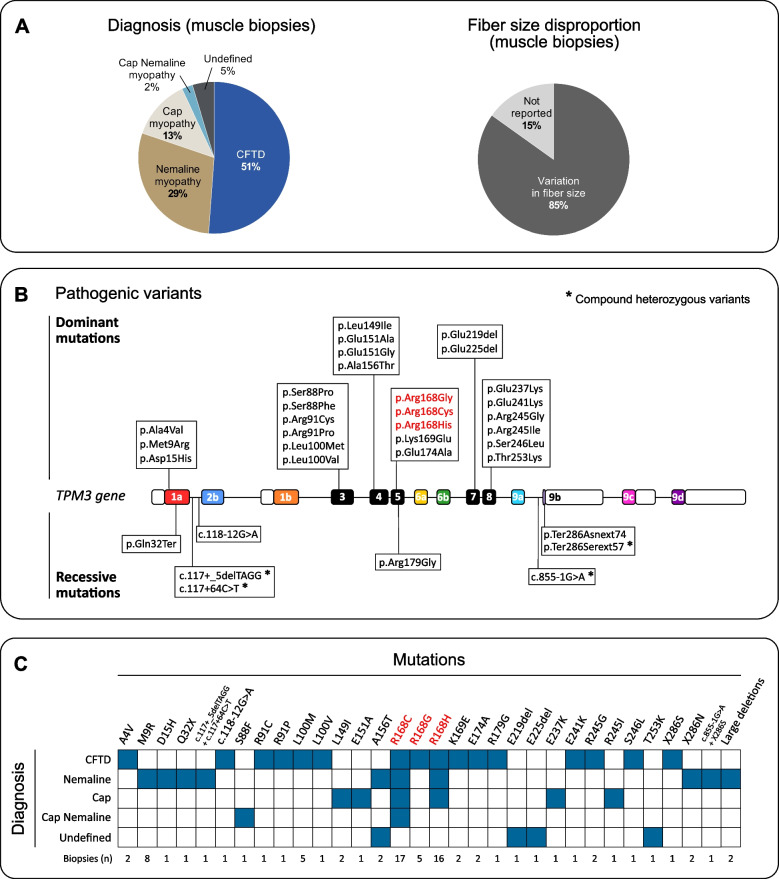
Histological and genetic basis of TPM3-related myopathy. **A** The assessment of distinct structural abnormalities in muscle histology has been historically used as a diagnostic tool for congenital myopathies. Congenital fiber type disproportion (CFTD) is the most common diagnosis in TPM3-related myopathy (51%), followed by nemaline myopathy (29%) and cap myopathy (13%). Both cap-like structures and nemaline rods can be simultaneously present in muscle biopsies (2%). In some biopsies, the diagnosis was classified as undefined (5%). Regardless of the diagnosis, variation in fiber size (also known as fiber size disproportion) with hypotrophy of type 1 muscle fibers was observed in most biopsies. These panels were generated based on *n* = 86 muscle biopsies from the literature. **B** A total of thirty-six TPM3 mutations either classified as pathogenic or likely pathogenic have been associated with the features of congenital myopathy in the literature. This represents 99 patients (*n* = 99) and 65 families (*n* = 65) documented (references [23, 28, 29, 43, 46, 88–126] as of August 2023). The p.Arg168 residue in exon 5 has been described as a mutational hotspot, documented in 41% of patients and in 30 families (colored in red). **C** The diagnosis (based on muscle biopsy) for each mutation is presented (colored in blue). There exists no clear genotype–histological correlation

The assessment of distinct structural abnormalities in muscle histology has been historically used as a diagnostic tool for congenital myopathies (for review, see [24]). In the mid-1990s, TPM3-related myopathy was first described as nemaline myopathy 1 (NEM1) in an Australian family [28, 29]. Since then, the identification of several patients and families affected by TPM3-related myopathy revealed a broader range of pathological features in muscle biopsies (Fig. 5A). As we write this review, congenital fiber type disproportion (CFTD) is the most common diagnosis made in TPM3 patients (51%). Congenital fiber type disproportion is characterized by the selective atrophy of type 1 muscle fibers, which are at least 12–25% smaller in diameter compared to type 2 fibers in the absence of other notable pathological findings [93, 128]. Nemaline myopathy represents the second most common diagnosis made in TPM3 patients (29%), characterized by the presence of nemaline rods, referred to as cytoplasmic inclusions in muscle fibers (seen with Gömöri trichrome staining) or electron-dense rod-shaped structures located at the Z-disk (seen with electron microscopy). Cap myopathy represents the third diagnosis made in TPM3 patients (13%), characterized by the presence of well-demarcated structures with reduced ATPase activity in subsarcolemmal regions, referred to as cap-like structures. Both nemaline rods and cap-like structures refer to protein aggregates essentially composed of the thin filament and Z-disk material and can be simultaneously observed in muscle biopsy (2%) (Fig. 5A) [23, 129].

The degree of protein aggregates observed in muscle biopsies and among patients with TPM3-related myopathy varies significantly, and there exists no evident correlation with disease severity. No clear relationship has also been established between the diagnosis and disease severity or disease onset as a distinct diagnosis can be made among family members affected by TPM3-related myopathy as well as in different muscle biopsies taken from a same individual [23, 88, 97, 101, 106]. In TPM3-related myopathy, the presence of protein aggregates is limited to small type 1 fibers and are often, if not always, associated with excessive fiber size variation (also known as fiber size disproportion). Additionally, fiber size variation is frequently reported regardless of the pathological diagnosis (85%) (Fig. 5A). Type 1 muscle fibers are on average 58% smaller in diameter compared to type 2 fibers [46]. This results from the selective hypotrophy of type 1 fibers, on average 0.6 times smaller than normal age-adjusted fibers, as well as from the selective hypertrophy of type 2 fibers, on average 1.6 times larger than normal age-adjusted fibers [88]. Fiber size variation is also commonly accompanied by the skewing of fiber type ratio (either type 1 predominance or type 2 predominance) [46]. However, no relationship between the degree of fiber size variation, fiber type predominance, and disease severity has been yet established as the degree of fiber type predominance can differ among family members as well as in different muscle biopsies taken from the same individual [46, 88, 93]. Slight increases in the content of internalized nuclei and the area of endomysial and perimysial fibrosis, as well as accumulation of mitochondria and glycogen particles were also sporadically observed [88, 90–92, 94, 95, 100, 101, 103, 106–109].

### The spectrum of TPM3 mutations associated with congenital myopathy

A total of 36 TPM3 mutations either classified as pathogenic or likely pathogenic have been associated with the features of congenital myopathy in the literature (for references, see [23, 28, 29, 43, 46, 88–126]) (Fig. 5B). This represents ninety-nine patients (*n* = 99) and 65 families (*n* = 65) documented since 1992. Open-source databases, patient registries, and variants of unknown significance (VUS) were not considered in this review, and therefore the number of patients affected by TPM3-related myopathy is likely to be underestimated.

Missense mutations represent the most common pathogenic variants identified in the *TPM3* gene, affecting approximately 88% of patients, followed by small in-frame deletions, large deletions, splice-site mutations, frameshift mutations and nonsense mutations. Additionally, compound heterozygous variants were documented in two patients [90, 94]. Nearly all pathogenic variants are located within exons specifically expressed by the striated Tpm3.12 isoform or commonly shared by both striated and cytoskeletal isoforms. So far, no pathogenic variants have been located in exons exclusively expressed by the cytoskeletal isoforms (exons 1b and 9c). Five variants are located in intronic regions, while two deletions span large genomic regions including exons 1–3 (large deletions are not depicted in Fig. 5B). The p.Arg168 residue in exon 5 has been described as a mutational hotspot, documented in 41% of patients and 30 families. The pattern of inheritance is predominantly autosomal dominant with a significant number of mutations arising spontaneously (de novo). An autosomal recessive pattern of inheritance only occurs in 10% of cases, often associated with a more severe phenotype [23]. Mutations leading to the absence of Tpm3.12 as well leading to truncated or enlarged protein are also associated with a more severe phenotype compared to missense mutations [23, 89–95, 125]. However, there exists no clear correlation between the type of mutations and diagnosis as patients carrying the same mutation can display different histological findings in muscle biopsy and vice versa (Fig. 5C).

## Disease mechanisms associated with TPM3-related myopathy

**Fig. 6:**
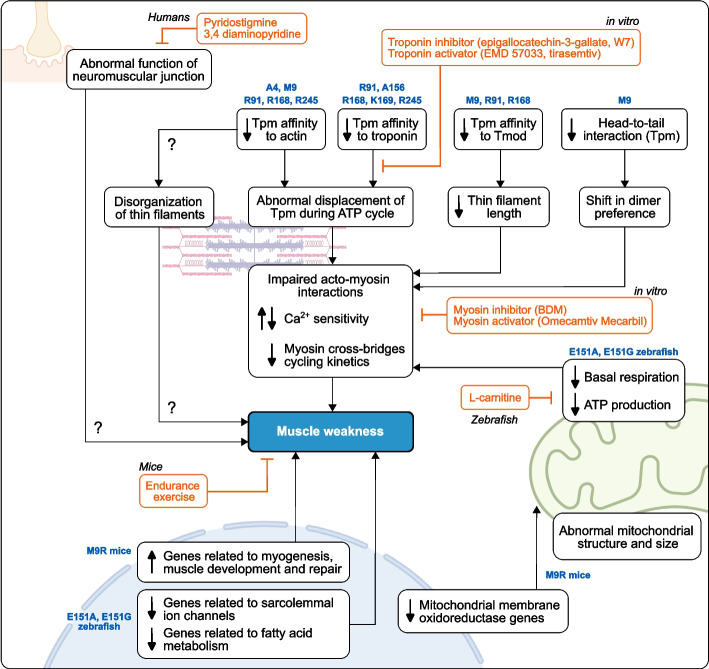
Molecular mechanisms associated with TPM3-related myopathy. Studies performed in reconstituted thin filaments in vitro and in patient muscle fibers showed that even minor changes within the tropomyosin structure can have major impacts on its function. Mutations in the *TPM3* gene (amino acid position are indicated in blue) can affect interaction between tropomyosin (Tpm) and actin, troponin, tropomodulin (Tmod) as well as other tropomyosin isoforms. These alterations can lead to thin filament dysfunction and impaired acto-myosin interaction during muscle contraction. Additionally, secondary disease mechanisms involving mitochondria, muscle development, ion channels and fatty acid metabolism are also depicted. Several therapeutic strategies have been developed to alleviate muscle weakness (shown in red). However, most of these strategies have not yet been tested in patients

The development of in vitro (e.g., reconstructed thin filaments) and in vivo model systems (e.g., TPM3^M9R^ mice, TPM3^E151A^ and TPM3^E151G^ zebrafish) as well as the use of patient muscle fibers have been essential to study the pathogenicity of TPM3 mutations and disease mechanisms. Some of these mechanisms have already been discussed (for review, see [23, 77, 130]). Here, we will discuss recent findings about the effects of TPM3 mutations on muscle function, providing new perspectives on the mechanisms that contribute to TPM3-related myopathy and the development of therapeutic strategies (Fig. 6).

### Impaired association between tropomyosin, actin, and troponin

Studies performed in reconstituted thin filaments in vitro and in patient muscle fibers showed that even minor changes within the tropomyosin structure can have major impacts on its function. Single amino acid substitutions, which represent the vast majority of pathogenic mutations in TPM3 patients, are distributed along the entire length of the Tpm3.12 molecule, with a significant portion of these substitutions found within the binding regions for actin, troponin, tropomodulin, and within the tropomyosin overlap junction [130]. Substitutions in conserved R91, R168, and R245 residues can disrupt the electrostatic contacts with D25 residue on actin, leading to a decreased affinity of tropomyosin for actin and reduced incorporation of Tpm3.12 mutant protein into actin filaments in muscle fibers [46, 131–134]. Substitutions located within the overlap junction such as A4V and M9R can also reduce this affinity (Fig. 6) [132, 135–137]. Additionally, substitutions such as R91P, A156T, R168H, R168G, K169E, and R245G can reduce the affinity of tropomyosin for troponin (Fig. 6) [138], while it has been suggested that A156T substitution can affect the binding between tropomyosin and myosin [139].

In striated muscle, tropomyosin function primarily relies on the cooperative interaction between tropomyosin, actin, troponin and myosin. Therefore, one can assume that the mechanisms leading to muscle weakness can be profoundly different depending on the site of mutation, but also the type of mutation. The association of tropomyosin with actin is made of weak but specific electrostatic interactions which enable tropomyosin to shift between regulatory states on the surface of the actin filament with a low energy cost [14]. A three-state model in which tropomyosin adopts three positions on actin-based thin filaments has been proposed as the basis for the steric regulation of muscle contraction (for review, see [10, 11]). (i) Blocked B-state (relaxed “off” state): in presence of the troponin complex and the absence of Ca^2+^, tropomyosin occludes myosin-binding sites on actin. (ii) Closed C-state: The binding of calcium to troponin C releases the pinning action of troponin I on tropomyosin location, allowing a 15° azimuthal shift of tropomyosin to partially expose myosin binding sites, and promote weak myosin binding to actin. (iii) Open M-state (activated “on” state): myosin head interaction then moves tropomyosin 10° further on actin to promote strong myosin binding and force production. Polarized fluorescence microscopy shows that several TPM3 mutations are associated with the abnormal displacement of tropomyosin towards incorrect conformational states throughout the ATP cycle (Fig. 6) [134, 139–142]. It has been hypothesized that freezing of tropomyosin towards the “off” state is associated with hypo-contractile phenotype (i.e., hypotonia, muscle weakness) [46, 131, 143]. In this context, several substitutions (e.g., L100M, R168C, R168G, R168H, K169E, R245G) have been associated with the reduction in myofilament Ca^2+^ sensitivity (pCa_50_) in muscle fibers from patients [46, 144]. In contrast, the relationship between the freezing of tropomyosin towards the “on” state and hyper-contractile phenotype (i.e., contractures, muscle stiffness) has been hypothesized for E219 and E225 deletions [92]. However, for several other mutations, there exists no such linear relationship. Substitutions R168H, E151A, and K169E have been associated with reduced or increased Ca^2+^ sensitivity in different studies [46, 131, 137, 140, 142, 144–146]. This may indicate the complexity of mature fibers in which the mechanical properties of myofilaments not only depend on tropomyosin but also on other factors which may have adapted to disease. For instance, fast α-actinin 3 and fast troponin T can be ectopically expressed in patient slow muscle fibers, which may partially explain phenotype discrepancy [43, 46]. Additionally, the use of different tropomyosin and troponin isoforms in reconstructed thin filaments and the use of different slow and fast myosin in in vitro motility assays can cause such discrepancies, while the effects of the mutations also highly depend on the dimer population used (homodimers vs. heterodimers) [137].

Altogether these studies show that TPM3 mutations can lead to reduced affinity of tropomyosin for actin and troponin resulting in abnormal displacement of the tropomyosin molecule throughout the ATP cycle (Fig. 6).

### Impaired myosin cross-bridges during contraction

The incorrect position of Tpm3.12 mutant protein can lead to less efficient and less stable transit of myosin along actin filaments during muscle fiber contraction [46, 92, 144, 145] (Fig. 6). A recent study suggests that substitutions associated with CFTD (e.g., E174A, R91P, E151A) increase the number of myosin heads in strong-binding state at low and high Ca^2+^ concentration [142]. In contrast, substitutions associated with cap myopathy (e.g., A156T) decrease the amount of myosin heads bound strongly to F-actin at high Ca^2+^ concentration [142]. However, this model presents some drawbacks and does not explain the disparities observed in patients carrying the same mutation (e.g., mutations on R168 residue). Immunostaining reveals that thin filament length is shorter in patient muscle fibers carrying the R168H mutation [144]. In this situation, fewer myosin binding sites would be exposed on the thin filaments and fewer myosin cross-bridges would be formed, limiting force production. In this study, force generation was depressed at long sarcomere lengths [144]. This profound change is caused by the reduction in length of the nebulin-free, Tmod4-capped pointed-end extension, while the length of the nebulin-stabilized core region of the thin filament was not affected [144]. The Arg168 residue is relatively far from the N-terminal segment of the tropomyosin molecule in which Tmod-binding region is located, and it remains unclear how R168H substitution can affect the polymerization and depolymerization of the thin filaments. In this context, the R91C substitution significantly decreases Tmod1 binding to Tpm3.12 and Tmod1 activity in reconstructed thin filaments in vitro [132]. Molecular dynamics (MD) simulation, a computational method that evaluates the motion of atoms and molecules, suggests that the reduced motion between Tpm3.12 and the thin filaments could perturb the binding of Tmod1 at the pointed-end [132]. The M9R substitution located in Tmod-binding region has been shown to reduce the helical content in the N-terminal segment of tropomyosin [18], leading to reduce affinity of tropomyosin for Tmod [43]. Unlike the R168H mutation, the M9R mutation leads to reduced force production at low sarcomere length in mice [147]. In contrast, A4V substitution does not interfere with the structure of the N-terminal segment and has little or no effect on Tmod binding and activity, suggesting a mutation-dependent mechanism [132].

Collectively, these studies suggest that TPM3 mutations can affect thin filament length via changes in binding affinity for Tmod and Tmod activity. Abnormal displacement of tropomyosin and reduced thin filament length are likely to cause changes in the number of myosin heads bound to the thin filaments, therefore affecting contractile properties (Fig. 6). However, these studies have been limited to very few mutations and such mechanisms need to be further explored.

### Skewing in tropomyosin dimer population

It has been shown that the M9R substitution destabilizes head-to-tail interaction between neighboring tropomyosin molecules and leads to the marked skewing in tropomyosin dimer population [43, 59, 137] (Fig. 6). This mutation abolishes the preferential formation of γβ heterodimers as well as the formation of γγ homodimers with both γ-chains being mutated. Instead, the pairing of γγ homodimers with only one γ-chain being mutated is predominantly found [43, 59, 137]. Because ββ homodimers are unstable under physiological conditions, the expression of β-tropomyosin (i.e., Tpm2.2) is drastically reduced in both mouse and human skeletal muscles carrying the M9R mutation [43, 46, 59]. Expression of β-tropomyosin is also reduced in deltoid muscle from a patient carrying a compound heterozygous variant in the *TPM3* gene. In this case, the X286S located within the overlap junction in the C-terminal segment of tropomyosin includes an additional 57 amino acids to the structure which may prevent dimerization [94]. However, the expression of β-tropomyosin was within normal range in quadriceps muscles from this patient, suggesting a muscle-dependent mechanism [145]. Other substitutions such as R91P, L100M, R168C, R168H, and E241K do not lead to a marked reduction in β-tropomyosin content in muscles, suggesting that the formation of γβ heterodimers is not significantly affected [46, 145]. However, such effects remain to be investigated in vitro since most of the studies have been conducted with homodimers (for recent discussion, see [77]). In conclusion, the switch of the tropomyosin dimer population appears to be a mutation-dependent and muscle-dependent mechanism and is likely to contribute to contractile dysfunction (Fig. 6).

Collectively, these studies indicate that TPM3 mutations have diverse effects on the structural and functional properties of tropomyosin, resulting in altered thin filament function and ultimately causing a decrease in force production in slow muscle fibers [46, 92, 144, 145] (Fig. 6).

### Animal models and secondary disease mechanisms

Animal models leveraged the discovery of secondary mechanisms related to TPM3-related myopathy. The transgenic *TPM3*^*M9R*^ mice, also referred to as HSA-αTm_slow_(Met9Arg) mice, recapitulate several features of the human disease for patients carrying this same mutation, such as an early formation of nemaline rods, an elevated content in oxidative muscle fibers, and late-onset muscle weakness [58]. In this model, disease severity varies significantly across muscle groups and is influenced by several factors including age, activity, and fiber type, rather than the expression of the mutant tropomyosin *per se* [58, 59, 147–151]. Additionally, skeletal muscles show significant disparities in the mechanisms underlying muscle weakness. For instance, reduced in vitro force production is associated with impairment in the level of myosin cross-bridges and increased Ca^2+^ sensitivity in gastrocnemius muscle fibers, while both cross-bridge and Ca^2+^ sensitivity levels are unaltered in EDL muscle fibers, suggesting that muscle weakness is likely governed by muscle-dependent mechanisms [151]. Microarray analysis reveals that the diaphragm exhibits the highest number of transcriptional changes compared to distal muscles which may indicate that the diaphragm is more profoundly affected in this model, concomitant with patients having reduced respiratory function and requiring mechanical ventilation while still being ambulant [150]. A prominent upregulation of genes related to myogenesis, muscle development, and myofiber immaturity is observed in several muscle groups (Fig. 6). In this context, it is suggested that the increased content in slow/fast oxidative fibers is due to an alteration in the maturation of muscle fibers rather than a defect in fiber type transition in TPM3^M9R^ mice. This finding was accompanied by an elevated number of centrally located nuclei positive fibers without signs of necrosis and degeneration, indicative of ongoing focal repair [150]. However, it is worth noting that muscle regeneration following stretch-induced muscle damage is associated with a significantly reduced number of fibers with central nuclei compared to wild-type mice, suggesting abnormal repair processes. Additionally, the diaphragm displayed a prominent downregulation of mitochondrial membrane oxidoreductase genes accompanied by a marked variation in the size and aberrant structure of mitochondria, as well as by the presence of lipofuscin inclusions indicative of oxidative stress [150]. Interestingly, basal mitochondrial respiration and ATP production are reduced in transgenic TPM3^E151A^ and TPM3^E151G^ zebrafish [113] (Fig. 6). This finding was associated with a reduction in swimming activity in both transgenic lines, with the E151G line being more severely affected. Additionally, the TPM3^E151G^ zebrafish line resembles congenital fiber type disproportion (CFTD), while TPM3^E151A^ zebrafish line resembles nemaline myopathy, suggesting that not only the site but also the type of substitution play an important role in TPM3-related myopathy. Bulk RNA sequencing performed in the E151G line shows a large downregulation of genes involved in anatomy structure development, cell differentiation, cell morphogenesis, ion channels, and fatty acid metabolism which may contribute to the pathogenesis [113] (Fig. 6).

### Therapeutic strategies

Currently, there is no cure for TPM3-related myopathy, and treatment is supportive and based on the individual's specific symptoms and needs. Physical therapy, occupational therapy, and speech therapy may be helpful in managing muscle weakness and feeding difficulties. Respiratory support, such as noninvasive ventilation or tracheostomy, may be necessary in individuals with severe respiratory insufficiency. Surgery may also be considered in some cases to address scoliosis.

However, several therapeutic strategies have been tested in the laboratory (Fig. 6). Pharmacological strategies targeting thin and thick filaments aim at reversing contractile dysfunction at the sarcomere level. For instance, the use of troponin activators (e.g., EMD 57033, tirasemtiv), troponin inhibitors (e.g., epigallocatechin-3-gallate, W7), myosin activator (e.g., Omecamtiv Mecarbil) and myosin inhibitor (e.g., BDM) partially reverse contractile defects in patient muscle fibers and reconstructed thin filaments in vitro [134, 140, 141, 144]. In these studies, diverse effects on contractile properties are observed depending on the strategy used and the mutation. Because TPM3 substitutions can have different effects on the behavior of tropomyosin, troponin, and myosin, it is likely that different pharmacological strategies will be required. However, the beneficial effect of such strategies is still unclear since these studies have been limited to a few substitutions (e.g., R91P, R168H, E174A) and have not been tested in vivo. Furthermore, compounds used in slow, type 1 muscle fibers target both cardiac and slow skeletal isoforms, and the effect on cardiac function in the context of TPM3-related myopathy is still unknown.

Pharmacological strategies targeting secondary mechanisms have also been tested (Fig. 6). For instance, altered muscle function, mitochondrial respiration, and molecular pathways were partially reversed in transgenic E151G zebrafish larvae with the use of L-carnitine [113]. It is hypothesized that L-carnitine can enhance long-chain fatty acyl-CoA into the mitochondria for β-oxidation to generate ATP to rescue muscle weakness. However, L-carnitine did not restore the swimming performance neither in adult zebrafish nor in transgenic E151A zebrafish. Furthermore, the use of tyrosine, terazosin, taurine, and creatine did not restore the swimming performance in both zebrafish lines [113]. Beneficial effect of L-carnitine in TPM3 patients has not been yet assessed. In contrast, the use of neuromuscular transmission-enhancing agents pyridostigmine and 3,4 diaminopyridine produced a modest increase in function in a 19-year-old patient carrying R168H mutation with abnormal function of neuromuscular junction [99]. This patient presented abnormal function of neuromuscular junction, yet salbutamol failed to produce any clinical benefit [99]. Myopathic EMG is a common feature observed in TPM3 patients. However, neuromuscular junctions have not been studied in this context, and such a therapeutic strategy to improve neuromuscular transmission has not been reported for TPM3 patients in other studies. Additionally, endurance exercise does not exacerbate muscle pathology in TPM3^M9R^ mice and can alleviate disuse-induced weakness [148, 149] (Fig. 6).

## Conclusion

In summary, several TPM3 isoforms are temporally and spatially expressed in developing and mature skeletal muscles. While great progress has been made in identifying these isoforms and understanding some of their functions, much work still needs to be done to completely elucidate the complexity of their different roles. In this context, the role of cytoskeletal isoforms in the pathogenesis of congenital myopathy is currently unknown. Therefore, new model systems need to be developed to better understand the multiple roles played by TPM3 within myofibers and disease. Additionally, these models will serve as valuable tools for facilitating efficient high-throughput drug screening and gene therapy testing. We anticipate that in the next decade, much progress will be made towards these goals and viable therapies will be developed for patients. We hope that this review will stimulate interest for future work focused on the function of all TPMs, including TPM1 and TPM2 in skeletal muscle and in myopathies.

## Data Availability

Not applicable.

## References

[1] Hitchcock-DeGregori SE, Barua B (2017). Tropomyosin structure, function, and interactions: a dynamic Regulator. Subcell Biochem.

[2] Manstein DJ, Meiring JCM, Hardeman EC, Gunning PW (2020). Actin–tropomyosin distribution in non-muscle cells. J Muscle Res Cell Motil.

[3] Geeves MA, Hitchcock-DeGregori SE, Gunning PW (2015). A systematic nomenclature for mammalian tropomyosin isoforms. J Muscle Res Cell Motil.

[4] Gunning P, O’Neill G, Hardeman E (2008). Tropomyosin-based regulation of the actin cytoskeleton in time and space. Physiol Rev.

[5] Gunning PW, Hardeman EC, Lappalainen P, Mulvihill DP (2015). Tropomyosin - master regulator of actin filament function in the cytoskeleton. J Cell Sci.

[6] Bailey K (1946). Tropomyosin: a new asymmetric protein component of muscle. Nature.

[7] Bailey K (1948). Tropomyosin: a new asymmetric protein component of the muscle fibril. Biochem J.

[8] Lin JJ, Lin JL (1986). Assembly of different isoforms of actin and tropomyosin into the skeletal tropomyosin-enriched microfilaments during differentiation of muscle cells in vitro. J Cell Biol.

[9] Handel SE, Greaser ML, Schultz E, Wang SM, Bulinski JC, Lin JJ (1991). Chicken cardiac myofibrillogenesis studied with antibodies specific for titin and the muscle and nonmuscle isoforms of actin and tropomyosin. Cell Tissue Res.

[10] Gordon A, Homsher E, Regnier M (2000). Regulation of contraction in striated muscle. Physiol Rev.

[11] Lehman W, Craig R (2008). Tropomyosin and the steric mechanism of muscle regulation. Adv Exp Med Biol.

[12] Billeter R, Heizmann CW, Reist U, Howald H, Jenny E (1981). α- and β-tropomyosin in typed single fibers of human skeletal muscle. FEBS Lett.

[13] Pieples K, Wieczorek DF (2000). Tropomyosin 3 increases striated muscle isoform diversity. Biochemistry.

[14] Li X, Tobacman LS, Mun JY, Craig R, Fischer S, Lehman W (2011). Tropomyosin position on F-actin revealed by EM reconstruction and computational chemistry. Biophys J.

[15] Pavadai E, Rynkiewicz MJ, Ghosh A, Lehman W (2020). Docking troponin t onto the tropomyosin overlapping domain of thin filaments. Biophys J.

[16] Jagatheesan G, Rajan S, Wieczorek DF (2010). Investigations into tropomyosin function using mouse models. J Mol Cell Cardiol.

[17] Lehman W, Pavadai E, Rynkiewicz MJ (2021). C-terminal troponin-I residues trap tropomyosin in the muscle thin filament blocked-state. Biochem Biophys Res Commun.

[18] Greenfield NJ, Fowler VM (2002). Tropomyosin requires an intact N-terminal coiled coil to interact with tropomodulin. Biophys J.

[19] Li XE, Orzechowski M, Lehman W, Fischer S (2014). Structure and flexibility of the tropomyosin overlap junction. Biochem Biophys Res Commun.

[20] Behrmann E, Müller M, Penczek PA, Mannherz HG, Manstein DJ, Raunser S (2012). Structure of the rigor actin-tropomyosin-myosin complex. Cell.

[21] Kostyukova AS (2007). Leiomodin/tropomyosin interactions are isoform specific. Arch Biochem Biophys.

[22] Marttila M, Hanif M, Lemola E, Nowak KJ, Laitila J, Grönholm M (2014). Nebulin interactions with actin and tropomyosin are altered by disease-causing mutations. Skelet Muscle.

[23] Marttila M, Lehtokari VL, Marston S, Nyman TA, Barnerias C, Beggs AH (2014). Mutation update and genotype-phenotype correlations of novel and previously described mutations in TPM2 and TPM3 causing congenital myopathies. Hum Mutat.

[24] North KN, Wang CH, Clarke N, Jungbluth H, Vainzof M, Dowling JJ (2014). Approach to the diagnosis of congenital myopathies. Neuromuscul Disord.

[25] Huang K, Bi FF, Yang H (2021). A systematic review and meta-analysis of the prevalence of congenital myopathy. Front Neurol.

[26] Pelin K, Wallgren-Pettersson C (2019). Update on the genetics of congenital myopathies. Semin Pediatr Neurol.

[27] Gineste C, Laporte J (2023). Therapeutic approaches in different congenital myopathies. Curr Opin Pharmacol.

[28] Laing NG, Majda BT, Akkari PA, Layton MG, Mulley JC, Phillips H (1992). Assignment of a gene (NEM1) for autosomal dominant nemaline myopathy to chromosome I. Am J Hum Genet.

[29] Laing NG, Wilton SD, Akkari PA, Dorosz S, Boundy K, Kneebone C (1995). A mutation in the α tropomyosin gene TPM3 associated with autosomal dominant nemaline myopathy. Nat Genet.

[30] Clayton L, Reinach FC, Chumbley GM, MacLeod AR (1988). Organization of the hTMnm gene. Implications for the evolution of muscle and non-muscle tropomyosins. J Mol Biol.

[31] Graham IR, Hamshere M, Eperon IC (1992). Alternative splicing of a human alpha-tropomyosin muscle-specific exon: identification of determining sequences. Mol Cell Biol.

[32] Beisel KW, Kennedy JE (1994). Identification of novel alternatively spliced isoforms of the tropomyosin-encoding gene, TMnm, in the rat cochlea. Gene.

[33] Dufour C, Weinberger RP, Schevzov G, Jeffrey PL, Gunning P (1998). Splicing of two internal and four carboxyl-terminal alternative exons in nonmuscle tropomyosin 5 pre-mRNA is independently regulated during development. J Biol Chem.

[34] Dube DK, Dube S, Abbott L, Elsekaily O, Sanger JW, Sanger JM (2020). Sarcomeric TPM3 expression in human heart and skeletal muscle. Cytoskeleton.

[35] Cunningham F, Allen JE, Allen J, Alvarez-Jarreta J, Amode MR, Armean IM (2022). Ensembl 2022. Nucleic Acids Res.

[36] Schevzov G, Whittaker SP, Fath T, Lin JJC, Gunning PW (2011). Tropomyosin isoforms and reagents. Bioarchitecture.

[37] Kee AJ, Schevzov G, Nair-Shalliker V, Robinson CS, Vrhovski B, Ghoddusi M (2004). Sorting of a nonmuscle tropomyosin to a novel cytoskeletal compartment in skeletal muscle results in muscular dystrophy. J Cell Biol.

[38] Hook J, Lemckert F, Qin H, Schevzov G, Gunning P (2004). Gamma tropomyosin gene products are required for embryonic development. Mol Cell Biol.

[39] Hook J, Lemckert F, Schevzov G, Fath T, Gunning P (2011). Functional identity of the gamma tropomyosin gene: Implications for embryonic development, reproduction and cell viability. BioArchitecture.

[40] Gunning P, Gordon M, Wade R, Gahlmann R, Lin CS, Hardeman E (1990). Differential control of tropomyosin mRNA levels during myogenesis suggests the existence of an isoform competition-autoregulatory compensation control mechanism. Dev Biol.

[41] Xi H, Langerman J, Sabri S, Chien P, Young CS, Younesi S (2020). A human skeletal muscle atlas identifies the trajectories of stem and progenitor cells across development and from human pluripotent stem cells. Cell Stem Cell.

[42] Muthuchamy M, Pajak L, Howles P, Doetschman T, Wieczorek DF (1993). Developmental analysis of tropomyosin gene expression in embryonic stem cells and mouse embryos. Mol Cell Biol.

[43] Ilkovski B, Mokbel N, Lewis RA, Walker K, Nowak KJ, Domazetovska A (2008). Disease severity and thin filament regulation in M9R TPM3 nemaline myopathy. J Neuropathol Exp Neurol.

[44] Jin Y, Peng Y, Lin Z, Chen Y-C, Wei L, Hacker TA (2016). Comprehensive analysis of tropomyosin isoforms in skeletal muscles by top-down proteomics. J Muscle Res Cell Motil.

[45] Oe M, Ojima K, Nakajima I, Chikuni K, Shibata M, Muroya S (2016). Distribution of tropomyosin isoforms in different types of single fibers isolated from bovine skeletal muscles. Meat Sci.

[46] Yuen M, Cooper ST, Marston SB, Nowak KJ, Mcnamara E, Mokbe N (2015). Muscle weakness in TPM3-myopathy is due to reduced Ca2+-sensitivity and impaired acto-myosin cross-bridge cycling in slow fibres. Hum Mol Genet.

[47] Bonnet A, Lambert G, Ernest S, Dutrieux FX, Coulpier F, Lemoine S (2017). Quaking RNA-binding proteins control early myofibril formation by modulating tropomyosin. Dev Cell.

[48] Marston SB, Copeland O, Messer AE, MacNamara E, Nowak K, Zampronio CG (2013). Tropomyosin isoform expression and phosphorylation in the human heart in health and disease. J Muscle Res Cell Motil.

[49] Peng Y, Yu D, Gregorich Z, Chen X, Beyer AM, Gutterman DD (2013). In-depth proteomic analysis of human tropomyosin by top-down mass spectrometry. J Muscle Res Cell Motil.

[50] Begue G, Raue U, Jemiolo B, Trappe S (2017). DNA methylation assessment from human slow- and fast-twitch skeletal muscle fibers. J Appl Physiol.

[51] Oe M, Ojima K, Muroya S (2021). Difference in potential DNA methylation impact on gene expression between fast- and slow-type myofibers. Physiol Genomics.

[52] Serrano AL, Murgia M, Pallafacchina G, Calabria E, Coniglio P, Lømo T (2001). Calcineurin controls nerve activity-dependent specification of slow skeletal muscle fibers but not muscle growth. Proc Natl Acad Sci U S A.

[53] Lin J, Wu H, Tarr PT, Zhang C-Y, Wu Z, Boss O (2002). Transcriptional co-activator PGC-1 alpha drives the formation of slow-twitch muscle fibres. Nature.

[54] Jackson HE, Ono Y, Wang X, Elworthy S, Cunliffe VT, Ingham PW (2015). The role of Sox6 in zebrafish muscle fiber type specification. Skelet Muscle.

[55] Petrany MJ, Swoboda CO, Sun C, Chetal K, Chen X, Weirauch MT (2020). Single-nucleus RNA-seq identifies transcriptional heterogeneity in multinucleated skeletal myofibers. Nat Commun.

[56] Dos Santos M, Backer S, Saintpierre B, Izac B, Andrieu M, Letourneur F (2020). Single-nucleus RNA-seq and FISH identify coordinated transcriptional activity in mammalian myofibers. Nat Commun.

[57] Hall MP, Nagel RJ, Fagg WS, Shiue L, Cline MS, Perriman RJ (2013). Quaking and PTB control overlapping splicing regulatory networks during muscle cell differentiation. RNA.

[58] Corbett MA, Robinson CS, Dunglison GF, Yang N, Joya JE, Stewart AW (2001). A mutation in alpha-tropomyosin(slow) affects muscle strength, maturation and hypertrophy in a mouse model for nemaline myopathy. Hum Mol Genet.

[59] Corbett MA, Anthony Akkari P, Domazetovska A, Cooper ST, North KN, Laing NG (2005). An alphatropomyosin mutation alters dimer preference in nemaline myopathy. Ann Neurol.

[60] Schevzov G, O’Neill G (2008). Tropomyosin gene expression in vivo and in vitro. Adv Exp Med Biol.

[61] Kee AJ, Yang L, Lucas CA, Greenberg MJ, Martel N, Leong GM (2015). An actin filament population defined by the tropomyosin Tpm3.1 regulates glucose uptake. Traffic.

[62] Matsuda R, Spector DH, Strohman RC (1983). Regenerating adult chicken skeletal muscle and satellite cell cultures express embryonic patterns of myosin and tropomyosin isoforms. Dev Biol.

[63] Heeley DH, Dhoot GK, Frearson N, Perry SV, Vrbova G (1983). The effect of cross-innervation on the tropomyosin composition of rabbit skeletal muscle. FEBS Lett.

[64] Heeley DH, Dhoot GK, Perry SV (1985). Factors determining the subunit composition of tropomyosin in mammalian skeletal muscle. Biochemical Journal.

[65] Pieples K, Arteaga G, Solaro RJ, Grupp I, Lorenz JN, Boivin GP (2002). Tropomyosin 3 expression leads to hypercontractility and attenuates myofilament length-dependent Ca(2+) activation. Am J Physiol Heart Circ Physiol.

[66] Lehman W, Hatch V, Korman V, Rosol M, Thomas L, Maytum R (2000). Tropomyosin and actin isoforms modulate the localization of tropomyosin strands on actin filaments. J Mol Biol.

[67] Fowler VM, Dominguez R (2017). Tropomodulins and leiomodins: actin pointed end caps and nucleators in muscles. Biophys J.

[68] Gokhin DS, Kim NE, Lewis SA, Hoenecke HR, D’Lima DD, Fowler VM (2012). Thin-filament length correlates with fiber type in human skeletal muscle. Am J Physiol Cell Physiol.

[69] Robaszkiewicz K, Śliwinska M, Moraczewska J (2020). Regulation of actin filament length by muscle isoforms of tropomyosin and cofilin. Int J Mol Sci.

[70] Kostyukova AS, Choy A, Rapp BA (2006). Tropomodulin binds two tropomyosins: a novel model for actin filament capping. Biochemistry.

[71] Chereau D, Boczkowska M, Skwarek-Maruszewska A, Fujiwara I, Hayes DB, Rebowski G (1979). Leiomodin is an actin filament nucleator in muscle cells. Science.

[72] Moroz NA, Novak SM, Azevedo R, Colpan M, Uversky VN, Gregorio CC (2013). Alteration of tropomyosin-binding properties of tropomodulin-1 affects its capping ability and localization in skeletal myocytes. J Biol Chem.

[73] Lewis RA, Yamashiro S, Gokhin DS, Fowler VM (2014). Functional effects of mutations in the tropomyosin-binding sites of tropomodulin1 and tropomodulin3. Cytoskeleton.

[74] Kuhn TB, Bamburg JR (2008). Tropomyosin and ADF/cofilin as collaborators and competitors. Adv Exp Med Biol.

[75] Agrawal PB, Joshi M, Savic T, Chen Z, Beggs AH (2012). Normal myofibrillar development followed by progressive sarcomeric disruption with actin accumulations in a mouse Cfl2 knockout demonstrates requirement of cofilin-2 for muscle maintenance. Hum Mol Genet.

[76] Janco M, Suphamungmee W, Li X, Lehman W, Lehrer SS, Geeves MA (2013). Polymorphism in tropomyosin structure and function. J Muscle Res Cell Motil.

[77] Matyushenko AM, Levitsky DI (2020). Molecular mechanisms of pathologies of skeletal and cardiac muscles caused by point mutations in the tropomyosin genes. Biochem Mosc.

[78] Matyushenko AM, Kleymenov SY, Susorov DS, Levitsky DI (2018). Thermal unfolding of homodimers and heterodimers of different skeletal-muscle isoforms of tropomyosin. Biophys Chem.

[79] Matyushenko AM, Shchepkin DV, Kopylova GV, Bershitsky SY, Levitsky DI (2020). Unique functional properties of slow skeletal muscle tropomyosin. Biochimie.

[80] Gateva G, Kremneva E, Reindl T, Kotila T, Kogan K, Gressin L (2017). Tropomyosin isoforms specify functionally distinct actin filament populations in vitro. Curr Biol.

[81] Sung LA, Lin JJC (1994). Erythrocyte tropomodulin binds to the N-Terminus of hTM5, a tropomyosin isoform encoded by the γ-tropomyosin gene. Biochem Biophys Res Commun.

[82] Kis-Bicskei N, Vig A, Nyitrai M, Bugyi B, Talián GC (2013). Purification of tropomyosin Br-3 and 5NM1 and characterization of their interactions with actin. Cytoskeleton.

[83] Bryce NS, Schevzov G, Ferguson V, Percival JM, Lin JJC, Matsumura F (2003). Specification of actin filament function and molecular composition by tropomyosin isoforms. Mol Biol Cell.

[84] Jansen S, Goode BL (2019). Tropomyosin isoforms differentially tune actin filament length and disassembly. Mol Biol Cell.

[85] Robaszkiewicz K, Ostrowska Z, Marchlewicz K, Moraczewska J (2016). Tropomyosin isoforms differentially modulate the regulation of actin filament polymerization and depolymerization by cofilins. FEBS J.

[86] Martin C, Schevzov G, Gunning P (2010). Alternatively spliced N-terminal exons in tropomyosin isoforms do not act as autonomous targeting signals. J Struct Biol.

[87] Vlahovich N, Kee AJ, Van der Poel C, Kettle E, Hernandez-Deviez D, Lucas C (2009). Cytoskeletal tropomyosin Tm5NM1 is required for normal excitation–contraction coupling in skeletal muscle. Pollard TD, editor. Mol Biol Cell.

[88] Clarke NF, Kolski H, Dye DE, Lim E, Smith RLL, Patel R (2008). Mutations in TPM3 are a common cause of congenital fiber type disproportion. Ann Neurol.

[89] Tan P, Briner J, Boltshauser E, Davis MR, Wilton SD, North K (1999). Homozygosity for a nonsense mutation in the alpha-tropomyosin slow gene TPM3 in a patient with severe infantile nemaline myopathy. Neuromuscul Disord.

[90] Pelin K, Sagath L, Lehtonen J, Kiiski K, Tynninen O, Paetau A (2023). Novel compound heterozygous splice-site variants in TPM3 revealed by RNA sequencing in a patient with an unusual form of nemaline myopathy: a case report. J Neuromuscul Dis..

[91] Yogev Y, Bistritzer J, Sadaka Y, Michaelovsky A, Cavari Y, Feinstein Y (2022). Transcript-based diagnosis and expanded phenotype of an intronic mutation in TPM3 myopathy. Mol Diagn Ther.

[92] Donkervoort S, Papadaki M, de Winter JM, Neu MB, Kirschner J, Bolduc V (2015). TPM3 deletions cause a hypercontractile congenital muscle stiffness phenotype. Ann Neurol.

[93] Lawlor MW, DeChene ET, Roumm E, Geggel AS, Moghadaszadeh B, Beggs AH (2010). Mutations of tropomyosin 3 (TPM3) are common and associated with type 1 myofiber hypotrophy in congenital fiber type disproportion. Hum Mutat.

[94] Wattanasirichaigoon D, Swoboda KJ, Takada F, Tong HQ, Lip V, Iannaccone ST (2002). Mutations of the slow muscle α-tropomyosin gene, TPM3, are a rare cause of nemaline myopathy. Neurology.

[95] Kiiski K, Lehtokari VL, Manzur AY, Sewry C, Zaharieva I, Muntoni F (2015). A Large Deletion Affecting TPM3, Causing Severe Nemaline Myopathy. J Neuromuscul Dis.

[96] Schreckenbach T, Schröder JM, Voit T, Abicht A, Neuen-Jacob E, Roos A (2014). Novel TPM3 mutation in a family with cap myopathy and review of the literature. Neuromuscul Disord.

[97] Kiphuth IC, Krause S, Huttner HB, Dekomien G, Struffert T, Schröder R (2010). Autosomal dominant nemaline myopathy caused by a novel α-tropomyosin 3 mutation. J Neurol.

[98] Citirak G, Witting N, Duno M, Werlauff U, Petri H, Vissing J (2014). Frequency and phenotype of patients carrying TPM2 and TPM3 gene mutations in a cohort of 94 patients with congenital myopathy. Neuromuscul Disord.

[99] Munot P, Lashley D, Jungbluth H, Feng L, Pitt M, Robb SA (2010). Congenital fibre type disproportion associated with mutations in the tropomyosin 3 (TPM3) gene mimicking congenital myasthenia. Neuromuscul Disord.

[100] Zheng Y, Lv H, Zhang W, Wang Z, Yuan Y (2020). Respiratory failure as the presenting symptom in a sporadic case of cap myopathy. J Neuropathol Exp Neurol.

[101] De Paula AM, Franques J, Fernandez C, Monnier N, Lunardi J, Pellissier JF (2009). A TPM3 mutation causing cap myopathy. Neuromuscul Disord.

[102] Bevilacqua JA, Contreras JP, Trangulao A, Hernández Ú, Brochier G, Díaz J (2022). Novel autosomal dominant TPM3 mutation causes a combined congenital fibre type disproportion-cap disease histological pattern. Neuromuscul Disord.

[103] Chen Z, Saini M, Koh JS, Lim GZ, Dang NJ, Prasad K (2023). A novel variant in the tropomyosin 3 gene presenting as an adult-onset distal myopathy - a case report. BMC Neurol.

[104] Perry L, Stimpson G, Singh L, Morrow JM, Shah S, Baranello G (2023). Muscle magnetic resonance imaging involvement patterns in nemaline myopathies. Ann Clin Transl Neurol.

[105] Park HJ, Jang H, Kim JH, Lee JH, Shin HY, Kim SM (2017). Discovery of pathogenic variants in a large Korean cohort of inherited muscular disorders. Clin Genet.

[106] Pénisson-Besnier I, Monnier N, Toutain A, Dubas F, Laing N (2007). A second pedigree with autosomal dominant nemaline myopathy caused by TPM3 mutation: A clinical and pathological study. Neuromuscul Disord.

[107] Almobarak S, Hu J, Langdon KD, Ang L, Campbell C (2021). α-tropomyosin gene (TPM3) mutation in an infant with nemaline myopathy. Clin Case Rep.

[108] Fidzianska A, Madej-Pilarczyk A, Hausmanowa-Petrusewicz I (2014). Is mutation p.Arg168Gly in TPM3 gene responsible for Type 1 fiber hypoplasia and cap structure formation?. Clin Neuropathol.

[109] Ohlsson M, Fidzianska A, Tajsharghi H, Oldfors A (2009). TPM3 mutation in one of the original cases of cap disease. Neurology.

[110] Ryan MM, Ilkovski B, Strickland CD, Schnell C, Sanoudou D, Midgett C (2003). Clinical course correlates poorly with muscle pathology in nemaline myopathy. Neurology.

[111] Stehlíková K, Skálová D, Zídková J, Haberlová J, Voháňka S, Mazanec R (2017). Muscular dystrophies and myopathies: the spectrum of mutated genes in the Czech Republic. Clin Genet.

[112] Yang Y, Muzny DM, Xia F, Niu Z, Person R, Ding Y (2014). Molecular findings among patients referred for clinical whole-exome sequencing. JAMA.

[113] Hsu P-J, Wang H-D, Tseng Y-C, Pan S-W, Sampurna BP, Jong Y-J (2021). l-Carnitine ameliorates congenital myopathy in a tropomyosin 3 de novo mutation transgenic zebrafish. J Biomed Sci.

[114] Durling HJ, Reilich P, Müller-Höcker J, Mendel B, Pongratz D, Wallgren-Pettersson C (2002). De novo missense mutation in a constitutively expressed exon of the slow alpha-tropomyosin gene TPM3 associated with an atypical, sporadic case of nemaline myopathy. Neuromuscul Disord.

[115] Klein A, Lillis S, Munteanu I, Scoto M, Zhou H, Quinlivan R (2012). Clinical and genetic findings in a large cohort of patients with ryanodine receptor 1 gene-associated myopathies. Hum Mutat.

[116] Natera-de Benito D, Ortez C, Jou C, Jimenez-Mallebrera C, Codina A, Carrera-García L (2021). The phenotype and genotype of congenital myopathies based on a large pediatric cohort. Pediatr Neurol.

[117] Tominaga K, Hayashi YK, Goto K, Minami N, Noguchi S, Nonaka I (2010). Congenital myotonic dystrophy can show congenital fiber type disproportion pathology. Acta Neuropathol.

[118] Fidziańska A (2002). “Cap disease”—a failure in the correct muscle fibre formation. J Neurol Sci.

[119] Waddell LB, Kreissl M, Kornberg A, Kennedy P, McLean C, Labarre-Vila A (2010). Evidence for a dominant negative disease mechanism in cap myopathy due to TPM3. Neuromuscul Disord.

[120] Gurgel-Giannetti J, Souza LS, Yamamoto GL, Belisario M, Lazar M, Campos W (2022). Nemaline myopathy in Brazilian patients: molecular and clinical characterization. Int J Mol Sci.

[121] Punetha J, Kesari A, Uapinyoying P, Giri M, Clarke NF, Waddell LB (2016). Targeted re-sequencing emulsion PCR panel for myopathies: results in 94 cases. J Neuromuscul Dis.

[122] Xu H, Liu H, Chen T, Song B, Zhu J, Liu X (2021). The R168G heterozygous mutation of tropomyosin 3 (TPM3) was identified in three family members and has manifestations ranging from asymptotic to serve scoliosis and respiratory complications. Genes Dis.

[123] Moreno CAM, de Paula Estephan E, Fappi A, Monges S, Lubieniecki F, Abath Neto OL (2020). Congenital fiber type disproportion caused by TPM3 mutation: a report of two atypical cases. Neuromuscul Disord.

[124] Gonzalez-Quereda L, Rodriguez MJ, Diaz-Manera J, Alonso-Perez J, Gallardo E, Nascimento A (2020). Targeted next-generation sequencing in a large cohort of genetically undiagnosed patients with neuromuscular disorders in Spain. Genes (Basel).

[125] Lehtokari V-L, Pelin K, Donner K, Voit T, Rudnik-Schöneborn S, Stoetter M (2008). Identification of a founder mutation in TPM3 in nemaline myopathy patients of Turkish origin. Eur J Hum Genet.

[126] Iglesias A, Anyane-Yeboa K, Wynn J, Wilson A, Truitt Cho M, Guzman E (2014). The usefulness of whole-exome sequencing in routine clinical practice. Genet Med.

[127] van Kleef ESB, van Doorn JLM, Gaytant MA, de Weerd W, Vosse BAH, Wallgren-Pettersson C (2022). Respiratory muscle function in patients with nemaline myopathy. Neuromuscul Disord.

[128] Clarke NF, North KN (2003). Congenital fiber type disproportion - 30 Years on. J Neuropathol Exp Neurol.

[129] Malfatti E, Schaeffer U, Chapon F, Yang Y, Eymard B, Xu R (2013). Combined cap disease and nemaline myopathy in the same patient caused by an autosomal dominant mutation in the TPM3 gene. Neuromuscul Disord.

[130] Moraczewska J (2020). Thin filament dysfunctions caused by mutations in tropomyosin Tpm3.12 and Tpm1.1. J Muscle Res Cell Motil.

[131] Marston S, Memo M, Messer A, Papadaki M, Nowak K, Mcnamara E (2013). Mutations in repeating structural motifs of tropomyosin cause gain of function in skeletal muscle myopathy patients. Hum Mol Genet.

[132] Moraczewska J, Robaszkiewicz K, Śliwinska M, Czajkowska M, Ly T, Kostyukova A (2019). Congenital myopathy-related mutations in tropomyosin disrupt regulatory function through altered actin affinity and tropomodulin binding. FEBS J.

[133] Gonchar AD, Kopylova GV, Kochurova AM, Berg VY, Shchepkin DV, Koubasova NA (2021). Effects of myopathy-causing mutations R91P and R245G in the TPM3 gene on structural and functional properties of slow skeletal muscle tropomyosin. Biochem Biophys Res Commun.

[134] Borovikov YS, Andreeva DD, Avrova SV, Sirenko VV, Simonyan AO, Redwood CS (2021). Molecular mechanisms of the deregulation of muscle contraction induced by the R90P mutation in Tpm3.12 and the weakening of this effect by BDM and W7. Int J Mol Sci.

[135] Moraczewska J, Greenfield NJ, Liu Y, Hitchcock-DeGregori SE (2000). Alteration of tropomyosin function and folding by a nemaline myopathy-causing mutation. Biophys J.

[136] Anthony Akkari P, Song Y, Hitchcock-DeGregori S, Blechynden L, Laing N (2002). Expression and biological activity of Baculovirus generated wild-type human slow α tropomyosin and the Met9Arg mutant responsible for a dominant form of nemaline myopathy. Biochem Biophys Res Commun.

[137] Matyushenko AM, Nefedova VV, Shchepkin DV, Kopylova GV, Berg VY, Pivovarova AV (2020). Mechanisms of disturbance of the contractile function of slow skeletal muscles induced by myopathic mutations in the tropomyosin TPM3 gene. FASEB J.

[138] Robaszkiewicz K, Ostrowska Z, Cyranka-Czaja A, Moraczewska J (2015). Impaired tropomyosin–troponin interactions reduce activation of the actin thin filament. Biochim Biophys Acta.

[139] Avrova SV, Karpicheva OE, Simonyan AO, Sirenko VV, Redwood CS, Borovikov YS (2019). The molecular mechanisms of a high Ca2+-sensitivity and muscle weakness associated with the Ala155Thr substitution in Tpm3.12. Biochem Biophys Res Commun.

[140] Karpicheva OE, Avrova SV, Bogdanov AL, Sirenko VV, Redwood CS, Borovikov YS (2023). Molecular mechanisms of deregulation of muscle contractility caused by the R168H mutation in TPM3 and its attenuation by therapeutic agents. Int J Mol Sci.

[141] Borovikov YS, Simonyan AO, Avrova SV, Sirenko VV, Redwood CS, Karpicheva OE (2020). Molecular Mechanisms of Muscle Weakness Associated with E173A Mutation in Tpm3.12. Troponin Ca2+ Sensitivity Inhibitor W7 Can Reduce the Damaging Effect of This Mutation. Int J Mol Sci.

[142] Borovikov Y, Karpicheva O, Simonyan A, Avrova S, Rogozovets E, Sirenko V (2018). The primary causes of muscle dysfunction associated with the point mutations in Tpm3.12; conformational analysis of mutant proteins as a tool for classification of myopathies. Int J Mol Sci.

[143] Memo M, Marston S (2013). Skeletal muscle myopathy mutations at the actin tropomyosin interface that cause gain- or loss-of-function. J Muscle Res Cell Motil.

[144] Ochala J, Gokhin DS, Penisson-Besnier I, Quijano-Roy S, Monnier N, Lunardi J (2012). Congenital myopathy-causing tropomyosin mutations induce thin filament dysfunction via distinct physiological mechanisms. Hum Mol Genet.

[145] Ottenheijm CAC, Lawlor MW, Stienen GJM, Granzier H, Beggs AH (2011). Changes in cross-bridge cycling underlie muscle weakness in patients with tropomyosin 3-based myopathy. Hum Mol Genet.

[146] Robaszkiewicz K, Dudek E, Kasprzak AA, Moraczewska J (2012). Functional effects of congenital myopathy-related mutations in gamma-tropomyosin gene. Biochim Biophys Acta Mol Basis Dis.

[147] De Haan A, Van der Vliet MR, Gommans IMP, Hardeman EC, Van Engelen BGM (2002). Skeletal muscle of mice with a mutation in slow α-tropomyosin is weaker at lower lengths. Neuromuscul Disord.

[148] Nair-Shalliker V, Kee AJ, Joya JE, Lucas CA, Hoh JF, Hardeman EC (2004). Myofiber adaptational response to exercise in a mouse model of nemaline myopathy. Muscle Nerve.

[149] Joya JE, Kee AJ, Nair-Shalliker V, Ghoddusi M, Nguyen MAT, Luther P (2004). Muscle weakness in a mouse model of nemaline myopathy can be reversed with exercise and reveals a novel myofiber repair mechanism. Hum Mol Genet.

[150] Sanoudou D, Corbett MA, Han M, Ghoddusi M, Nguyen MAT, Vlahovich N (2006). Skeletal muscle repair in a mouse model of nemaline myopathy. Hum Mol Genet.

[151] Gineste C, Ottenheijm C, Le Fur Y, Banzet S, Pecchi E, Vilmen C (2014). Alterations at the cross-bridge level are associated with a paradoxical gain of muscle function in vivo in a mouse model of Nemaline myopathy. PLoS One.

